# Synthesis of highly crystalline LaFeO_3_ nanospheres for phenoxazinone synthase mimicking activity†

**DOI:** 10.1039/d1ra02295d

**Published:** 2021-05-17

**Authors:** Mohamed Khairy, Abdelrahman H. Mahmoud, Kamal M. S. Khalil

**Affiliations:** Chemistry Department, Faculty of Science, Sohag University 82524 Egypt kms_khalil@yahoo.co.uk mohamed.khairy@science.sohag.edu.eg +20 (2) 01092099116

## Abstract

LaFeO_3_ nanospheres with an orthorhombic perovskite structure were synthesized by a sol–gel autocombustion method in the presence of different citric acid ratios (*x* = 2, 4, 8, and 16) and utilized for the photocatalytic conversion of *o*-aminophenol (OAP) to 2-aminophenoxazine-3-one (APX) for the first time. OAP is one of the most toxic phenolic derivatives used as a starting material in many industries; however, the dimerization product APX has diverse therapeutic properties. Photocatalytic conversion was carried out in ethanol/water and acetonitrile/water mixtures in the absence and presence of molecular oxygen at ambient temperature *via* the oxidative coupling reaction that mimics phenoxazinone synthase-like activity. The LaFeO_3_ samples showed a superior photocatalytic activity of OAP to APX with rate constants of 0.43 and 0.92 min^−1^ in the absence and presence of molecular oxygen, respectively. Thus, the LaFeO_3_ nanozymes could be used as promising candidates in industrial water treatment and phenoxazinone synthase-like activity.

## Abstract 2

LaFeO_3_ nanospheres were synthesized by a facile sol–gel autocombustion method and explored for the photocatalytic transformation of *o*-aminophenol to 2-aminophenoxazine-3-one.

## Graphical abstract

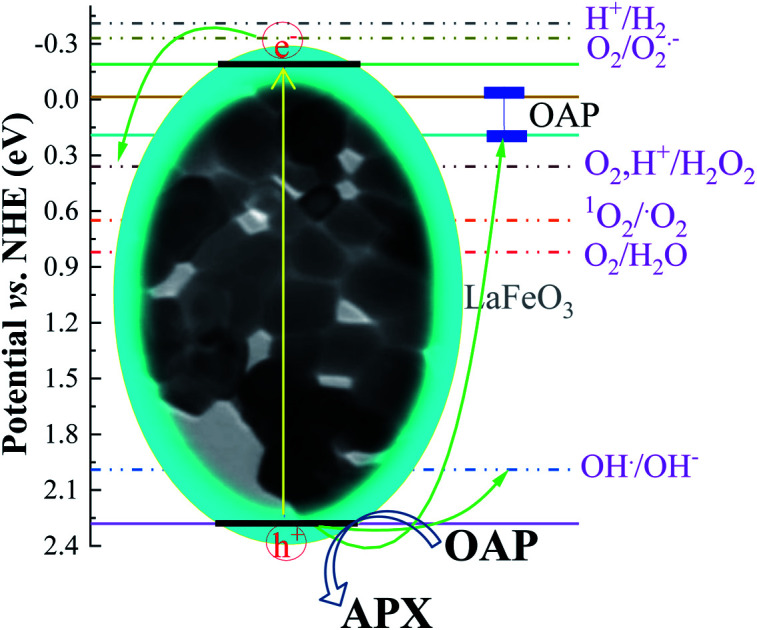

## 1. Introduction

Millions of tons of organics are intensively consumed in food additives, pharmaceuticals, pesticides, painting, dying, textile industries, and agricultural activities every year. Most of these undesired reagents or starting materials, particularly those called persistent contaminants, are subsequently discharge in the environment and cause serious health risks.^1^ Considering the environmental safety issues and energy-effective cost, many restrictions are assessed recently on chemical industries to switch and utilize alternative or greener manufacturing routes. However, large amounts of persistent organic contaminants are still discharged. Biological, thermal, physical, and chemical treatment technologies have been adopted for the mitigation of organics in the last decades.^2–4^ Several microorganisms are being utilized to degrade biodegradable organics but the process usually takes a long retention time. Thermal treatment also consumes a large quantity of energy. Membrane separations and adsorption are widely used for removing organic contaminants.^5,6^ Functional materials with large surface areas should be engineered and developed. However, a long separation time and surface regeneration with stable functionality are still major obstacles. Among the aforementioned technologies, chemical oxidation is a promising process for large-scale wastewater treatment under mild conditions. It is usually carried out using chemical reagents, which might be homogenous or heterogeneous processes. Thus, many research works have been devoted to develop both homogenous and heterogeneous catalysts for efficient wastewater treatment.^7,8^

The photocatalytic oxidation process is an efficient and eco-friendly methodology that utilizes light energy to drive the oxidation reaction, leading to the degradation of organic contaminants to low molecular oxygenated chemical species or the synthesis of new compounds.^9,10^ Irradiation of a semiconductor with a light stimulates the transfer of electrons in the valence band (VB) to the conduction band, leaving behind a positive charge carrier known as a hole.^11^ These photo-generated charge carriers (e^−^/h^+^) might be recombined again in the bulk catalyst nanoparticles, producing thermal energy or migrating to the catalyst surface. The surface charge carriers can further participate in the catalytic reaction through their redox potentials. The photo-generated hole acts as an electron acceptor in the oxidation processes. Alternatively, the surface adsorbed species such as H_2_O molecules and OH^−^ group may trap the photo-generated holes, producing hydroxyl radicals (˙OH), which is a non-selective strong oxidizing species with a redox potential of 2.80 V (*vs.* NHE). On the other hand, the electrons in the conduction band might reduce the adsorbed O_2_ molecules and form superoxide O_2_˙^−^ radicals. Metal oxides such as TiO_2_ and ZnO have been commonly used as heterogeneous photocatalysts.^12^ However, their wide energy band gap requires a high photo-energy for excitation. Regrettably, UV irradiation represents only less than 5% of solar energy, which limits these metal oxides' applicability. Accordingly, various attempts have been undertaken to make the photocatalysis process more efficient and economical by utilizing a wide range of visible-light energy.

Iron-based materials have been explored as visible-light-driven photocatalysts such as iron oxides,^13,14^ iron oxyhydroxide,^15^ SrFeO_3−*x*_,^16^ LaFeO_3_, and BiFeO_3_.^17^ Among several iron-based nanomaterials, LaFeO_3_ nanoparticles (NPs) have received much attention because of their thermal and chemical stability and unique optoelectronic properties. They have been utilized in many applications such as chemical sensors,^18^ fuel cells,^19^ water splitting,^20^ and photocatalysis.^21^ They have an orthorhombic structure with FeO_6_ octahedron units where La^3+^ lies between these units. LaFeO_3_ NPs has been used as a visible light-driven catalyst with a narrow band gap of about 2.0–2.6 eV; however, the photocatalytic efficiency is limited. The photocatalytic activity of LaFeO_3_ NPs was improved by (i) doping with other non-precious metals, *i.e.*, La_1−*x*_Sr_*x*_FeO_3_,^22^ La_1−*x*_Sb_*x*_FeO_3_,^23^ and La_1−*y*_Sr_*y*_Ni_1−*x*_Fe_*x*_O_3_,^24^ or (ii) immobilization on a suitable porous support, *i.e.*, LaFeO_3_/montmorillonite nanocomposites (LaFeO_3_/MMT),^25^ LaFeO_3_/silica composite,^26^ and LaFeO_3_/g-C_3_N_4_.^27^ Besides, different synthetic methods such as microwave-assisted method,^28^ co-precipitation,^29^ hydrothermal,^30^ thermal decomposition,^31^ combustion process,^32^ and sol–gel have been used.^33^ Despite the intensive efforts made to enhance the photocatalytic efficiency of LaFeO_3_ NPs, it is still low.

Phenol and its derivatives are widely used in several industrial applications such as manufacturing of dyes, explosives, paints, pharmaceuticals, pesticides, and herbicides. As a result, a large amount of phenolic compounds are discharged in water supplies. *o*-Aminophenol (OAP) is one of the most common phenolic derivatives, which is used as a starting material in many industries.^34^ It is a toxic reducing agent that causes toxic methemoglobinemia in human erythrocytes. It has oscillatory behavior because it can oxidize oxyhemoglobin and reduce methemoglobin *via* the oxidative coupling of two OAP molecules and forms 2-aminophenoxazinone-3-one.^35^ Iron and copper oxidase phenoxazinone synthase catalyzes the oxidative dimerization of OAP in the biosynthetic pathway of antibiotic Actinomycin D, which is used clinically in the treatment of specific types of cancers. Bioinspired catalysts based on manganese,^36^ cobalt,^37^ and copper complexes have been developed with oxidase-mimicking activity.^38^ Although these catalysts showed good catalytic activity, they are difficult to reuse. To the best of our knowledge, LaFeO_3_ NPs are widely used for the photo-degradation of organic pollutants but it is not reported as a photo-nanozyme for phenoxazinone synthase mimicking activity.

Herein, LaFeO_3_ NPs were synthesized by the controlled sol–gel autocombustion method using different citric acid ratios (*x* = 2, 4, 8, and 16). The LaFeO_3_ samples were characterized by transmission electron microscopy (TEM), nitrogen adsorption/desorption isotherm, X-ray diffraction (XRD), Fourier transform infrared spectroscopy (FTIR), and solid-state UV-Vis spectroscopy. Then, the catalytic activity of the LaFeO_3_ samples was explored for the conversion of OAP to APX dimer as an oxidase-mimicking photo-nanozyme in nitrogen and oxygen atmosphere at ambient temperature. The LaFeO_3_ NPs shows high photocatalytic activity for the conversion of OAP pollutants to a hand-safe and valuable product without losing their efficiency even after several reuse cycles.

## 2. Experimental

### 2.1. Chemicals

All chemicals were of the highest analytical grade and used without further purification. Lanthanum nitrate hexahydrate (La(NO_3_)_3_·6H_2_O), ferric nitrate nanohydrate Fe(NO_3_)_3_·9H_2_O, and citric acid C_6_H_8_O_7_ were supplied by Alpha Chemical Co. Ltd. Ammonium hydroxide (NH_4_OH) and OAP were purchased from BDH Chemicals Ltd. Hydrochloric acid and sodium hydroxide were purchased from Sigma Aldrich Co. Ltd.

### 2.2. Fabrication of LaFeO_3_ samples

The LaFeO_3_ samples were synthesized by a controlled sol–gel autocombustion method using different citric acid ratios *x* = 2, 4, 8, and 16. A stoichiometric amount of La(NO_3_)_3_·9H_2_O and Fe(NO_3_)_3_·6H_2_O was dissolved in distilled water under continuous magnetic stirring at room temperature to get a transparent solution. A particular citric acid concentration was introduced to the above mixture until a homogeneous sol was formed. To stabilize the formed sol, the pH of the mixture was adjusted to pH = 7 by the addition of ammonium hydroxide and left under vigorous stirring for one hour at room temperature. The dried sol was transferred to a clean crucible and combusted using a benzene stove. The resultant fluffy powder was collected and annealed at 500 °C for one hour with a heating rate of 1 °C min^−1^. The LaFeO_3_ samples were labeled as LFCx, where *x* represents the citric acid ratio and the calcination temperature, respectively.

### 2.3. Characterization of the LaFeO_3_ samples

The surface morphology of the LaFeO_3_ samples was determined by the transmission electron microscope JEOL JEM model 2100F. The LaFeO_3_ samples were grinded using mortar, dispersed in ethanol, dropped on a copper grid, and left to dry in an oven at 50 °C. The LaFeO_3_-supported grid was inserted into the TEM column using a CCD camera at 200 kV.

The wide-angle X-ray diffraction of the LaFeO_3_ samples was performed using D8-advance with monochromatic Cu K_α_ radiation (*λ* = 1.5418 Å) in the 2*θ* range from 4° to 80° with a scan rate of 0.05° min^−1^. The diffraction patterns were analyzed using PDF-2 Release 2009.

Nitrogen adsorption/desorption isotherms were obtained using a BELsorp mini, Japan at 77 K. The LaFeO_3_ samples were degassed by N_2_ gas at 300 °C for 8 hours. The pore size distribution and specific surface area was analyzed by the Barrett–Joyner–Halenda (BJH) and Brunauer–Emmett–Teller (BET) equations, respectively.

The UV-Vis diffuse reflectance spectra and spectrophotometric measurements of OAP were carried out in the range of 200–800 nm using the UV/Vis/NIR model JASCO 770 V. Fourier-transform infrared (FTIR) spectroscopy was studied in the range of 400–4000 cm^−1^ and performed using a Bruker Alpha II.

### 2.4. Photo-nanozyme activity

The batch photocatalytic experiments were carried out at pH 7 containing 3% acetonitrile or ethanol to dissolve OAP and to investigate the reaction mechanism. 10 mg of LaFeO_3_ was dispersed in a 50 mL quartz reactor perfectly closed with a rubber septum at room temperature. For inert conditions, the reactor was purged with N_2_ gas for fifteen minutes under continuous magnetic stirring to remove oxygen. Then, OAP was injected by syringe into the reaction medium to have a final solution of 0.5 mM OAP. The reactor was placed horizontally in front of a 75 W xenon arc lamp, producing a wide range of wavelengths to simulate one sun with the intensity of 100 mW cm^−2^. Prior to exposing the reactor to the xenon lamp, the reaction mixture was stirred for one hour in dark to reach adsorption/desorption equilibrium. At regular intervals, 2 mL of the reaction mixture was withdrawn and centrifuged for UV/Vis spectroscopy measurements at *λ* = 430 nm, which corresponds to *C*_t_. At the end of the reaction, the absorbance was measured and considered as *C*_∞_. The photocatalytic experiments were repeated three times to confirm the obtained results.

## 3. Results and discussion

### 3.1. Morphological and structural features of the LaFeO_3_ catalyst

**Fig. 1:**
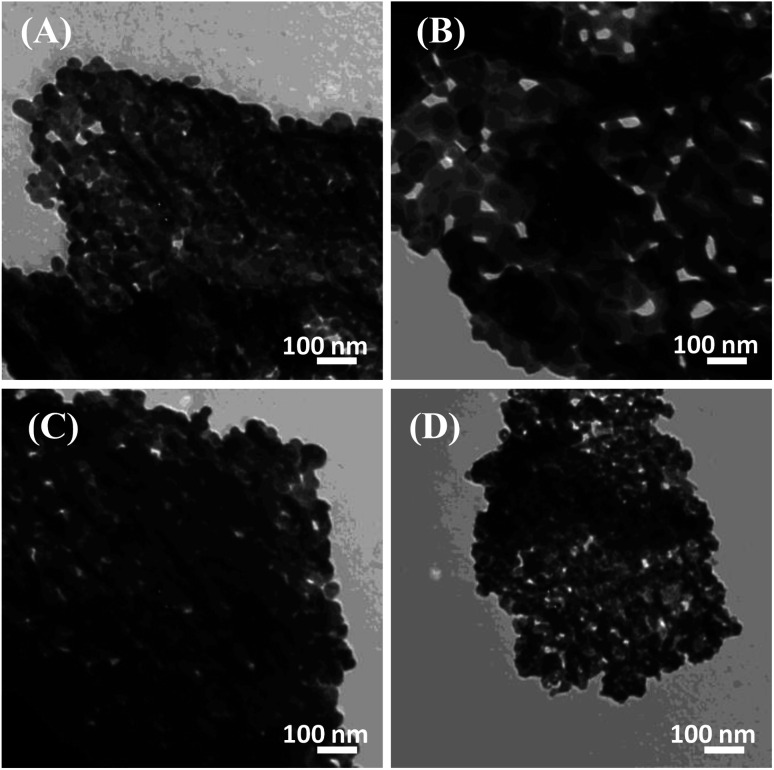
TEM micrographs of the LaFeO_3_ samples synthesized in the presence of different citric acid ratios of (A) 2, (B) 4, (C) 8, and (D) 16 annealed at 500 °C.

The control over the particle size was made evident using the controlled sol–gel autocombustion synthesis of LaFeO_3_ NPs. Fig. 1A–D illustrates the TEM micrographs of the LaFeO_3_ samples synthesized in the presence of different citric acid ratios. Aggregated LaFeO_3_ nanoparticles with spherical shape are shown with average sizes of 38, 70, 33, and 19 nm for the citric acid ratios of 2, 4, 8, and 16, respectively. The largest particle size was observed using the citric acid ratio of 4. Thus, the particle size of LaFeO_3_ could be controlled by optimizing the complexing agent ratio since an excess of citric acid retards large-size crystal formation.

**Fig. 2:**
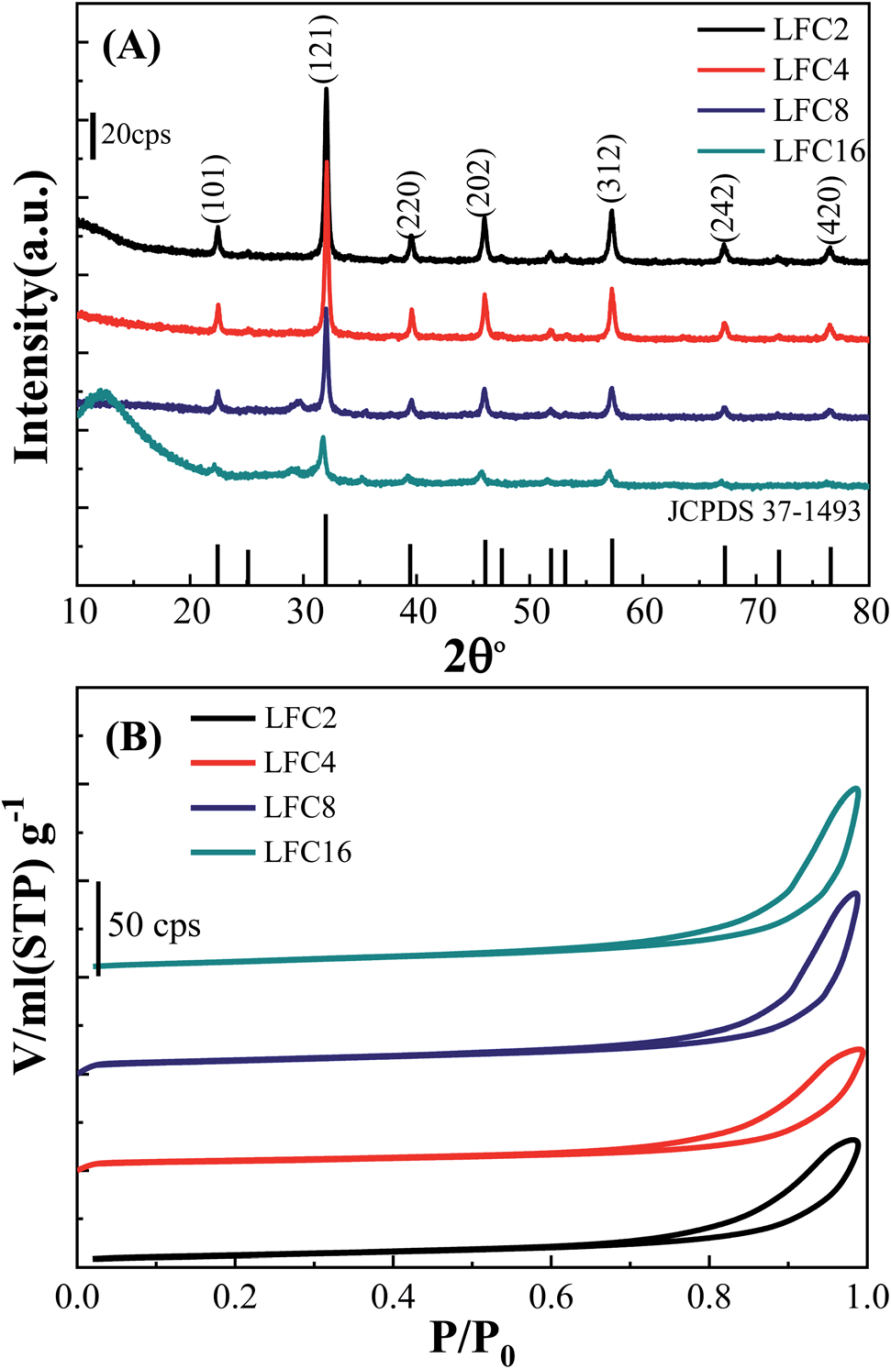
(A) XRD and (B) N_2_ adsorption/desorption isotherms of LaFeO_3_ samples synthesized *via* the sol–gel autocombustion method using different citric acid ratios.

Fig. 2A represents the XRD patterns of the LaFeO_3_ samples in the range of 2*θ* range of 10°–80°. Typical diffraction peaks of LaFeO_3_ samples were observed at 2*θ*° = 22.38°, 32.03°, 39.54°, 46.03°, 57.26°, 67.20°, and 76.47° corresponding to (101), (121), (220), (202), (312), (242), and (420), respectively. These diffraction peaks can be assigned to the orthorhombic crystal lattice of JCPDS 37-1493 with a space group *Pnma* and lattice parameters *a* = 5.566 Å, *b* = 7.854 Å, and *c* = 5.553 Å. No additional secondary peaks were observed regarding Fe_2_O_3_ or La_2_O_3_, indicating the complete conversion of the precursors to the pure crystalline LaFeO_3_ perovskite structure. The highest diffraction peaks were observed using a citric acid ratio of 4. However, Thirumalairajan *et al.* reported the increase in the citric acid ratio up to 3, wherein impurities such as La_2_O_3_ could be formed at 600 °C.^39^ The crystal size of the LaFeO_3_ samples was calculated using the Debye–Scherrer equation and found to be 26, 30, 27, and 20 nm. The crystal size of LaFeO_3_ increases from a citric acid ratio 2 to 4 and then reduces to the ratios of 8 and 16. These results agree with the TEM micrographs (Fig. 1). Thus, the citric acid ratios could be considered as a crucial prerequisite to form highly crystalline LaFeO_3_ nanoparticles with controlled crystallite sizes.

The textural properties of the LaFeO_3_ samples upon variation of the citric acid ratio were explored by measuring the N_2_ adsorption/desorption isotherms (Fig. 2B). The LaFeO_3_ samples showed a type IV isotherm with an H_3_ hysteresis loop at a relatively high pressure that featured a mesoporous network. The mesoporous network was built by the interconnection of LaFeO_3_ nanoparticles, as shown in the TEM micrographs (Fig. 1). The specific surface areas (*S*_BET_) of the LaFeO_3_ samples were derived by the Brunauer–Emmett–Teller method and found to be 21, 17, 24, and 28 m^2^ g^−1^ for citric acid ratio of *x* = 2, 4, 8, and 16, respectively. The specific surface area of LFC16 is almost three times higher than those prepared by the hydrothermal method.^39^ Thus, the high-surface area of the LaFeO_3_ samples were produced *via* a controlled facile sol–gel autocombustion method and it was interesting to investigate their photocatalytic efficiency.

**Fig. 3:**
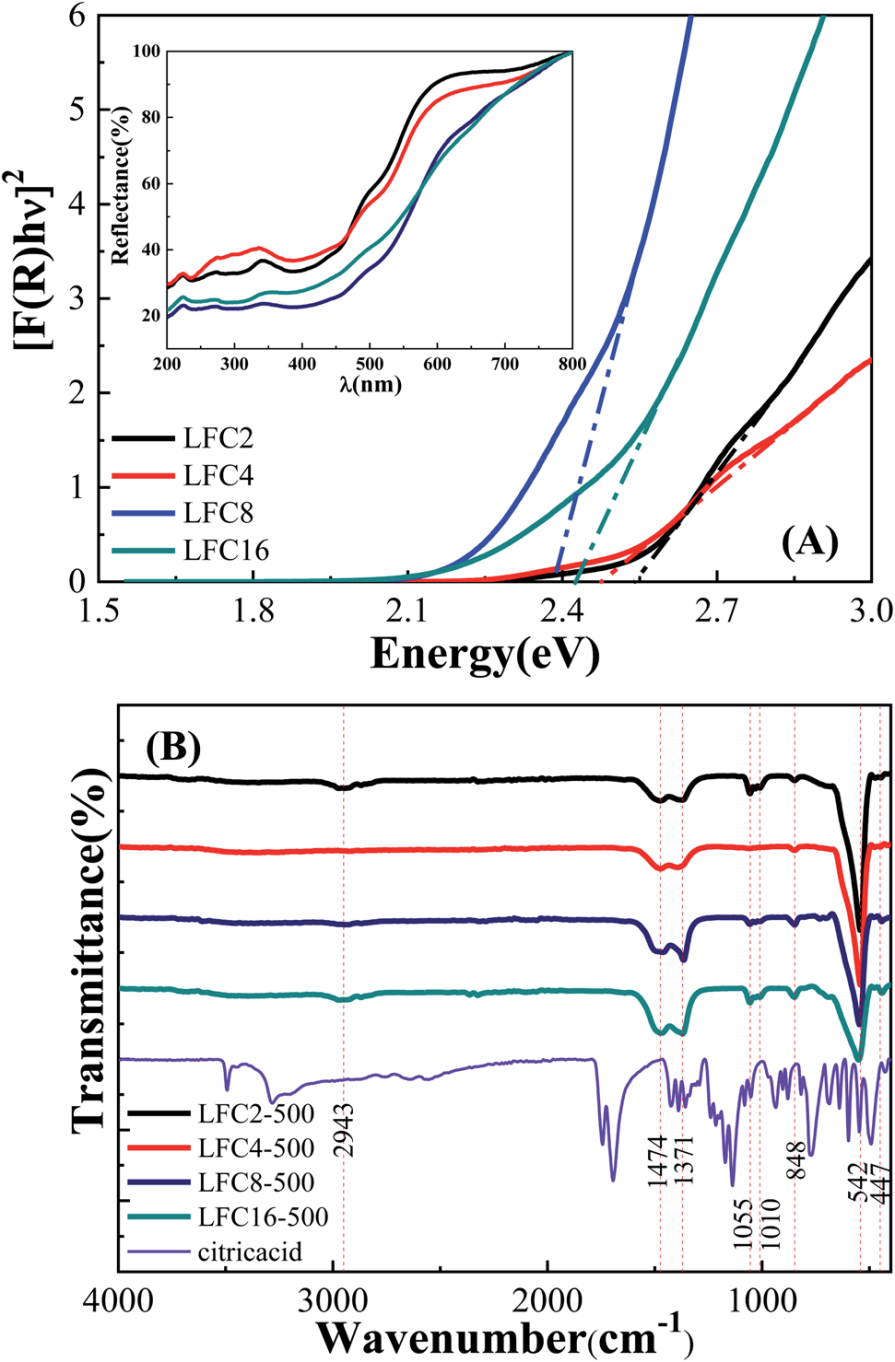
(A) Diffuse reflectance (% *R*) spectra and Kubelka–Munk curves, and (B) FTIR spectra of the LaFeO_3_ samples synthesized *via* the sol–gel autocombustion method by using different citric acid ratios.

The optical band gap of the LaFeO_3_ samples synthesized using different citric acid ratios was calculated using diffuse reflectance UV/Vis spectroscopy, as shown in Fig. 3A. The absorption edge of the LaFeO_3_ samples was found at *λ* > 600 where *R*% values increase gradually and reach almost 100% in this region. This absorption band is attributed to the electronic transition from O_2p_ → Fe_3d_ of the valance to the conduction band. This observation indicates that the LaFeO_3_ samples could serve as a visible-light-driven photocatalyst. The optical band gap energy (*E*_g_) was calculated based on the direct allowed transition (*n* = 2) of the linear segment of relationship [*F*(*R*) × *hν*]^2^*vs. hν* and was found to be 2.53, 2.47, 2.38, and 2.43 eV for citric acid ratio *x* = 2, 4, 8, and 16, respectively.^40^ The positions of the valence band (VB) and the conduction band (CB) edges were also measured at the point of zero charge by the following equation.^41^*E*_CB_ = *χ* − *E*_e_ − 0.5*E*_g_where *E*_CB_ is the potential of the conduction band edge, *χ* is the absolute Mulliken electronegativity of the constituent elements of the semiconductor, *E*_e_ is the energy of free electron on the hydrogen scale (∼4.5 eV). The *χ* value of LaFeO_3_ is about 5.54 eV (S1 in ESI†). The potential of the conduction band edge (*E*_CB_) was estimated to be −0.22, −0.19, −0.15, and −0.17 eV *vs.* NHE for LFC2, LFC4, LFC8, and LFC16, respectively. Besides, the potential of the valence band edge was calculated by; *E*_VB_ = *E*_CB_ + *E*_g_ and found to be 2.31, 2.28, 2.23, and 2.26 eV *vs.* NHE for LFC2, LFC4, LFC8, and LFC16, respectively.

The functionality of the LaFeO_3_ samples was analyzed by FTIR spectroscopy in the range 400–4000 cm^−1^, as shown in Fig. 3B. A small absorption peak followed by a sharp one was observed at 447 and 542 cm^−1^, which is characteristic of octahedral FeO_6_ and is assigned to O–Fe–O bending modes and Fe–O stretching vibration, respectively.^39^ These peaks enhanced in the citric acid ratios of 2 and 4 and suppressed for citric acid 8 and 16. These results are in good agreement with their XRD patterns since the crystallinity of ratios 2 and 4 is higher than that of 8 and 16. A small absorption peak at 848 cm^−1^ might be attributed to CH_2_ rocking. The absorption peaks at 1010 and 1055 cm^−1^ might relate to C–O and C–OH stretching.^42^ The absorption peaks at 1371 and 1475 cm^−1^ might also be attributed to CH_3_ and CH_2_ bending. The broad bands in the range of 2830–2950 cm^−1^ might be assigned to symmetric CH_2_ and asymmetric stretching of C–H.^43–45^ Interestingly, the peaks at 848, 1010, 1055, and 2950 cm^−1^ almost disappeared at the citric acid ratio of 4. Moreover, the characteristic absorption bands of C–O at 1371 and 1473 cm^−1^ showed a gradual enhancement by increasing the citric acid molar ratios.^46^ We can conclude that the functional carbon content increases in high citric acid ratios. It is also worthy to find that the presence of a high citric acid ratio reduces the particle size, crystallinity, and band gap and increases the surface area due to porous residual carbon. The functionality variation of LaFeO_3_ particles could induce different physical features and offer interesting pathways in the catalytic oxidation of phenols.^43^ Further, the high surface area also facilitates the diffusion of the species to the active centers and enhances the photocatalytic process.

### 3.2. Catalytic reactivity of the LaFeO_3_ samples

**Fig. 4:**
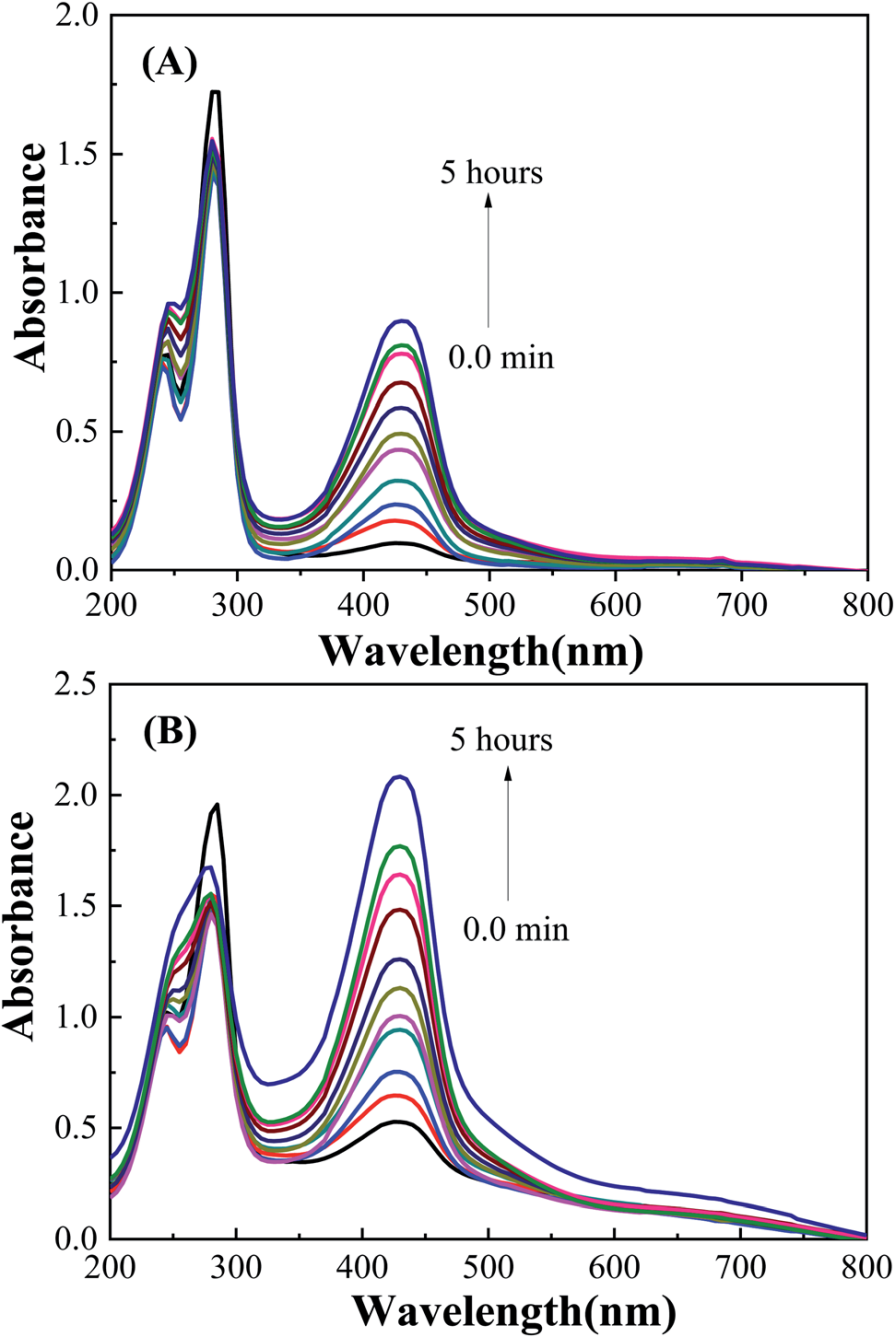
UV/Vis spectra for the photocatalytic conversion of OAP over (A) LFC4 and (B) LFC16 samples in ethanol/water mixture solution under nitrogen atmosphere.

**Fig. 5:**
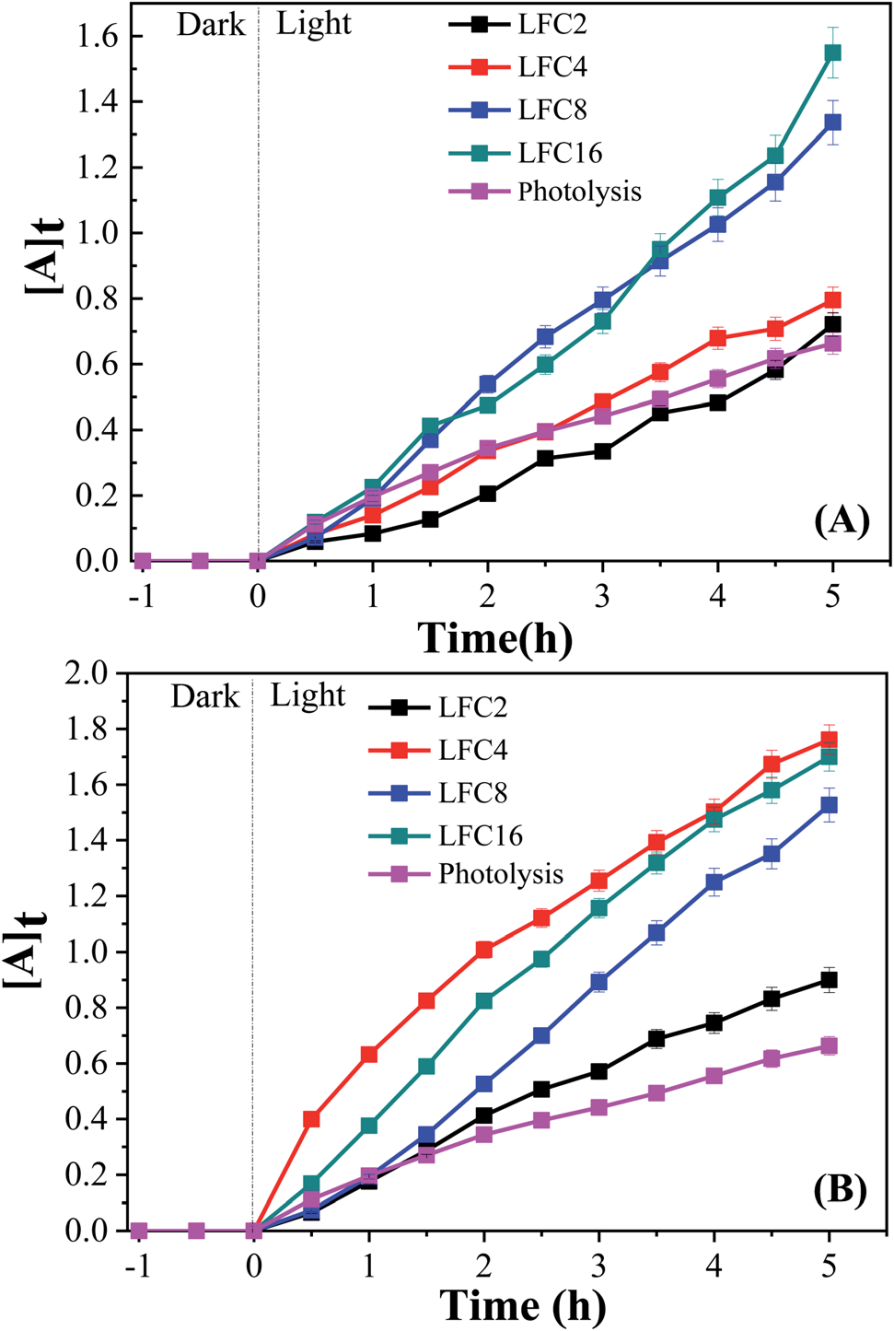
Analysis of absorbance time dependence in (A) ethanol/water under nitrogen atmosphere and (B) acetonitrile/water under an inert atmosphere.

The photocatalytic activity of the LaFeO_3_ samples was explored for the chemical transformation of OAP (toxic phenolic compound) to aminophenoxazinone-3-one (nontoxic) *via* the oxidase-like activity. The photocatalytic activity toward the oxidation of OAP molecules was estimated by measuring the absorbance at *λ* = 430 nm for aminophenoxazinone-3-one (APX) as a function of time. To investigate the impact of the citric acid ratio on the photocatalytic transformation of OAP, 0.01 g of LaFeO_3_ samples were introduced into a cylindrical reactor in the absence and presence of molecular oxygen. Fig. 4A and B shows the UV/Vis spectra for the photocatalytic conversion of OAP over LaFeO_3_ samples synthesized using citric acid ratios of 4 and 16 in ethanol/water mixture in a nitrogen atmosphere under solar irradiation. The absorbance spectral values at *λ* = 430 nm are attributed to APX increase with time. Fig. 5A and B shows the analysis of the absorbance values [*A*]_*t*_ with time. In the absence of LaFeO_3_, the absorbance values were found to be 0.66 and 0.66 in ethanol and acetonitrile mixtures after five hours of irradiation, respectively. Thus, the photo-oxidation (photolysis) of OAP in the absence of LaFeO_3_ was not affected by the solvent type. In the presence of 10 mg of LaFeO_3_ samples, the recorded absorbance values are 0.72, 0.79, 1.34, and 1.53 for LFC2, LFC4, LFC8, and LFC16 in ethanol/water mixture, respectively (Fig. 5A). The absorbance peak attributed to APX is enhanced by increasing the citric acid ratios. The LFC16 sample shows the highest conversion efficiency. Thus, the photocatalytic transformation is controlled by the amount of functionalized carbon residue around the LaFeO_3_ nanoparticles. To investigate the effect of the calcination temperature, the photocatalytic experiment was performed using LaFeO_3_ samples calcined at 300, 500, 600, and 700 °C as shown in Fig. S2 and S3.† No obvious change in the catalytic conversion rate of OAP to APX was observed by raising the annealing temperatures. To identify the oxidized products of OAP, mass spectrometry was carried out, as shown in Fig. S4.† OAP could be oxidized to form the dimeric product APX [C_12_H_11_N_2_O_2_].

On the other hand, the photocatalytic experiment was carried out in the presence of acetonitrile/water mixture. The analysis of the absorbance values as a function of time was found to be 0.9, 1.76, 1.53, and 1.7 for LFC2, LFC4, LFC8, and LFC16 samples after five hours of irradiation, respectively. Noticeably, there was a slight improvement in the photocatalytic activity for the LFC2, LFC8, and LFC16 samples. However, a large enhancement in the photocatalytic transformation of OAP was observed for LFC4 (Fig. 5B). These results indicate that the selection of the solvent is a crucial factor for the photocatalytic transformation of OAP to APX using LaFeO_3_ nanoparticles because it might dominate the separation of charge carriers (electrons/holes).

The effect of the solution pH on the photocatalytic conversion of OAP to APX is also investigated, as shown in Fig. S5.† The photocatalytic conversion was carried out in a nitrogen atmosphere using acetonitrile/water mixture. 10 mg LFC4 and 0.5 mM OAP were introduced to the reactor under light irradiation and the absorbance was estimated as a function of time. The highest conversion efficiency was observed in solutions of pH 5 and 7. Therefore, pH 7 was used in further experiments. In order to postulate the photocatalytic conversion pathway of OAP, EDTA and *t*-butyl alcohol (TB) were used as the hole and hydroxyl radical scavenger, respectively. 2 mmol EDTA or TB were added to the photocatalytic reactor containing 10 mg LFC4 and 0.5 mM OAP in the acetonitrile/water mixture, as shown in Fig. S6.† The photocatalytic activity of the LaFeO_3_ sample was significantly suppressed in the presence of EDTA or TB, indicating that the hole and hydroxyl radical play a vital role in the photocatalytic conversion of OAP.

**Scheme 1:**
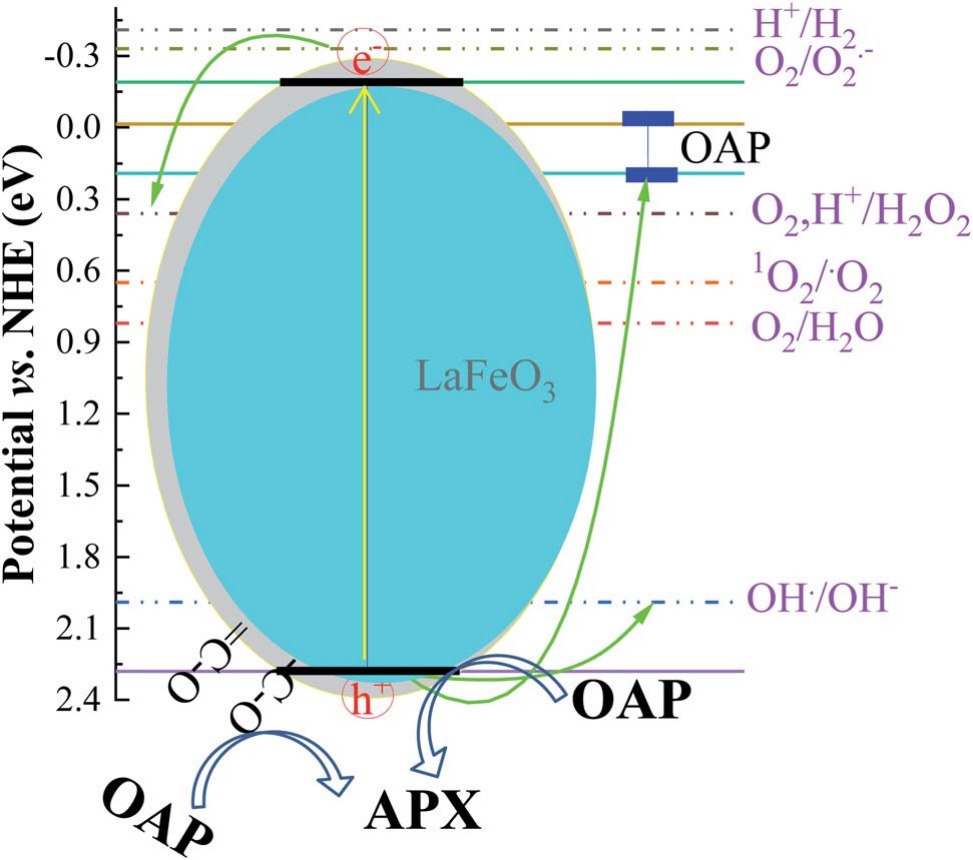
Photocatalytic conversion mechanism of OAP to APX over LaFeO_3_ at pH 7.

The absorption of solar irradiation by LaFeO_3_ semiconductor materials leads to the transfer of the electrons (e^−^) in the valence band to a higher energy level known as the conduction band, in which they can move loosely in the crystal lattice, leaving behind a positive charge carrier known as a hole (h^+^). The photo-generated charge carriers (e^−^/h^+^) can further participate in the catalytic processes through redox reactions of reactive species or recombined in the bulk (Scheme 1 and eqn (1)). The photo-generated hole in the valence band (at *E*_VB_ = 2.28 eV) is an electron acceptor and can oxidize phenolic species or water molecules (at *E*_˙OH/H_2_O_ = 1.99 eV) to form highly reactive hydroxyl radical (˙OH) at pH 7, as presented in eqn (2). Then, the photo-generated holes or hydroxyl radicals oxidize OAP to APX since the photocatalytic activity is suppressed in the presence of EDTA and TB molecules (eqn (3)–(7)). Simultaneously, the conduction band works as an electron donor and thus reduces the contaminant or other chemical species present in the reaction media. However, the electrons in the conduction band are not able to reduce the oxygen molecules (*E*_O_2_/˙O_2__ = −0.33 eV). In presence of air, the oxygen molecules might be reduced to hydrogen peroxide (*E*_O_2_,H^+^/H_2_O_2__ = 0.36 eV), which is able to oxidize OAP to APX.

1 LaFeO_3_ + *hν* → e^−^ +h^+^

2 H_2_O + h^+^ → ˙OH + H^+^

3
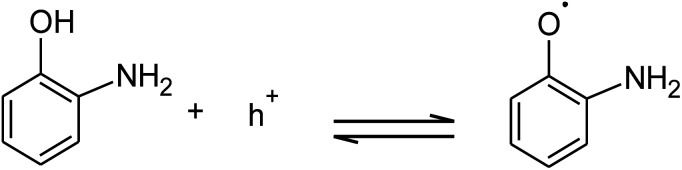
4
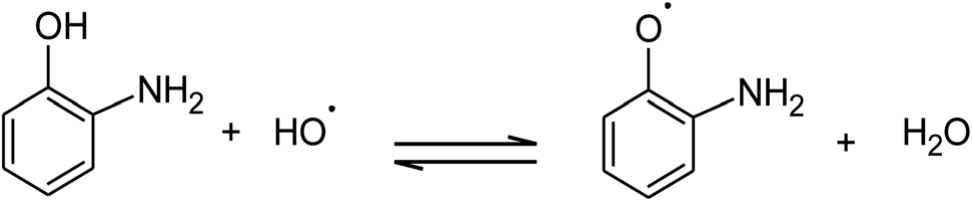
5
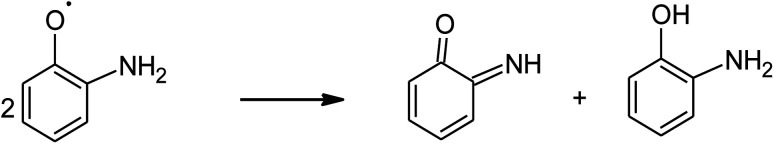
6
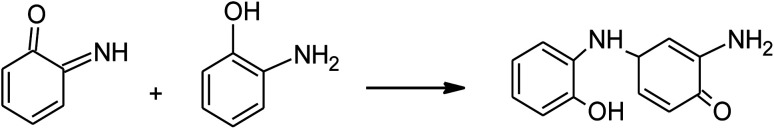
7
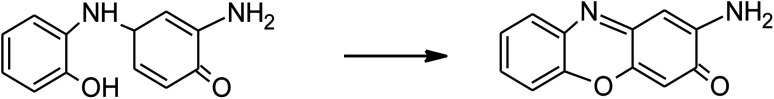


In the presence of ethanol, the photocatalytic efficiency was reduced because ethanol acts as a competitive scavenger of photo-generated holes and hydroxyl radicals. The surface photo-generated holes and resultant hydroxyl radicals are consumed by ethanol and are no longer available. The photocatalytic efficiency becomes the highest using LFC4 in the acetonitrile/water mixture. Interestingly, the presence of functionalized residual carbon in the sample LFC16 enhanced the photocatalytic conversion efficiency. To explore the effect of functional carbon in the LaFeO_3_ samples, the catalytic conversion of OAP was studied in the dark, as shown in Fig. S7.† LFC16 shows the highest catalytic efficiency compared to the LFC2 and LFC4 samples, indicating that functionalized carbon residue acts as an oxidative center for the catalytic conversion of OAP to APX.^44^ Under solar irradiation, OAP molecules with a size 6 Å are competitively diffused and adsorbed onto the oxygenated carbon surface, particularly on LFC16, and scavenge the photo-generated holes and the produced hydroxyl radicals even in the presence of ethanol.^45^ The carbon residue might also reduce the recombination rate of the photo-generated charge carriers.^46^ Thus, the sample LFC16 offers bimodal oxidative centers as the photo-generated holes related to LaFeO_3_ and oxygenated functional carbon. These oxidative centers enhance the photocatalytic conversion of OAP to APX.

**Fig. 6:**
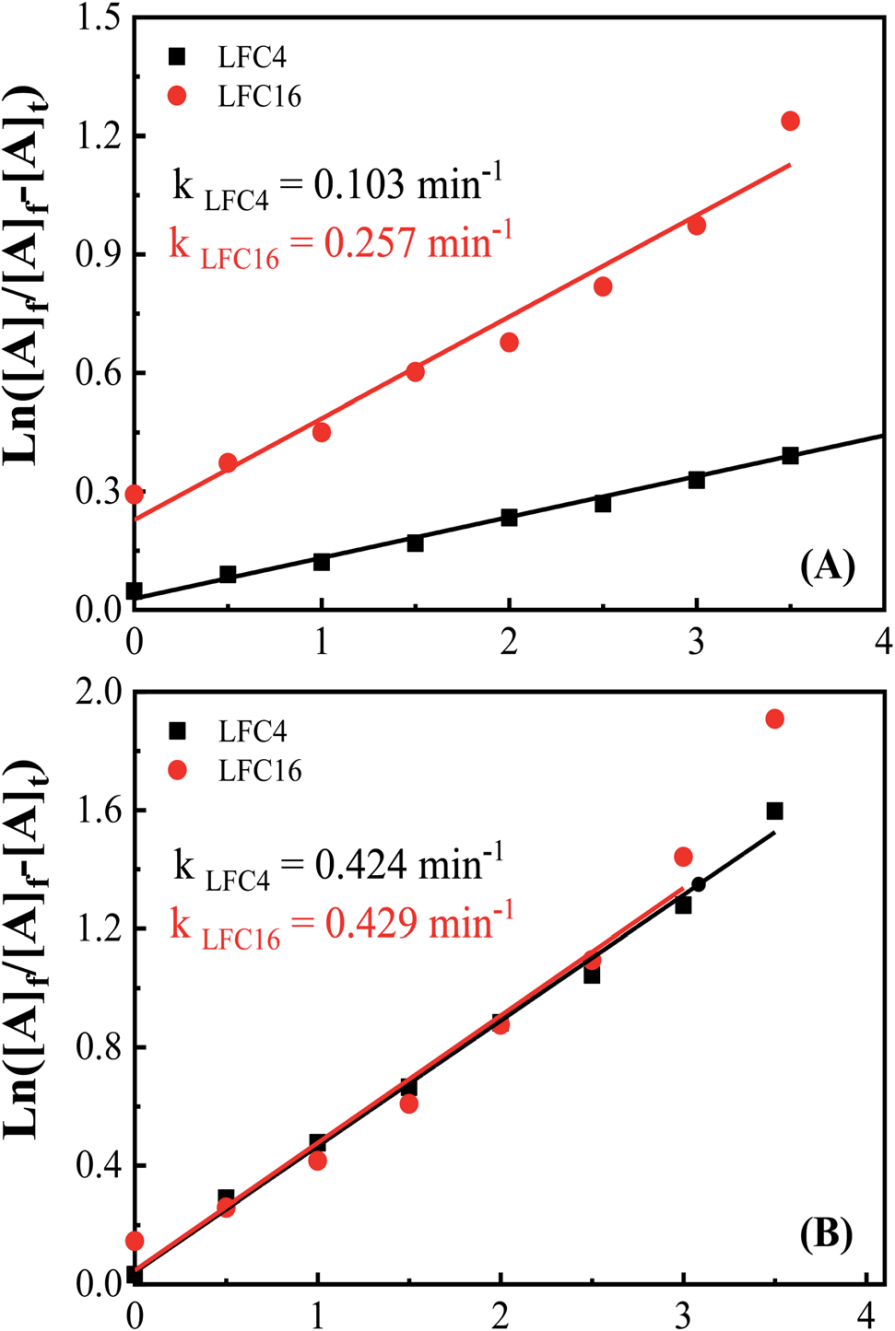
Integrated first-order kinetics for the photocatalytic oxidation of OAP in (A) ethanol/water under nitrogen atmosphere and (B) acetonitrile/water under nitrogen atmosphere.

To explore the difference between the LaFeO_3_ samples and their ability for the photocatalytic oxidation of OAP, electrochemical impedance spectroscopy (EIS) was carried out in 0.1 M Na_2_SO_4_ at *E*_dc_ of 0.5 V (*vs.* Ag/AgCl), and an excitation AC voltage of 10 mV peak-to-peak in the frequency range of 100 kHz to 3 HZ was employed. Fig. S8† shows the Nyquist diagrams of fluorine-doped tin oxide (FTO) substrate modified with LaFeO_3_ samples in the absence and presence of OAP under dark and light conditions. Typical semicircles regarding charge transfer resistance were observed for all the LaFeO_3_ samples. The analysis of the EIS is given in Table S8.† The charge transfer resistance (*R*2) generally decreases under solar irradiation and LFC4 shows a lower value. Thus, sample LFC4 will be sensitive to light irradiation among the LaFeO_3_ samples. LFC2 also shows a large Warburg impedance under light irradiation because of the aggregation of LaFeO_3_ nanoparticles, which might suppress the diffusion of OAP to trap the hole or ˙OH. Further, LFC8 and LFC16 show a significant decrease in the Warburg impedance under light irradiation due to the diffusion of OAP to the active centers in porous carbon networks. Thus, the photo-generated charge carriers might be trapped into the carbon network that are able to oxidize the diffused OAP in the presence of ethanol or acetonitrile (Fig. 5A and B). Fig. 6A and B shows the integrated first-order equation with respect to OAP concentrations. The heterogeneous rate constant was calculated to be 0.103 and 0.257 min^−1^ in ethanol/water and 0.424 and 0.429 min^−1^ in acetonitrile/water for LFC4 and LFC16, respectively.

**Fig. 7:**
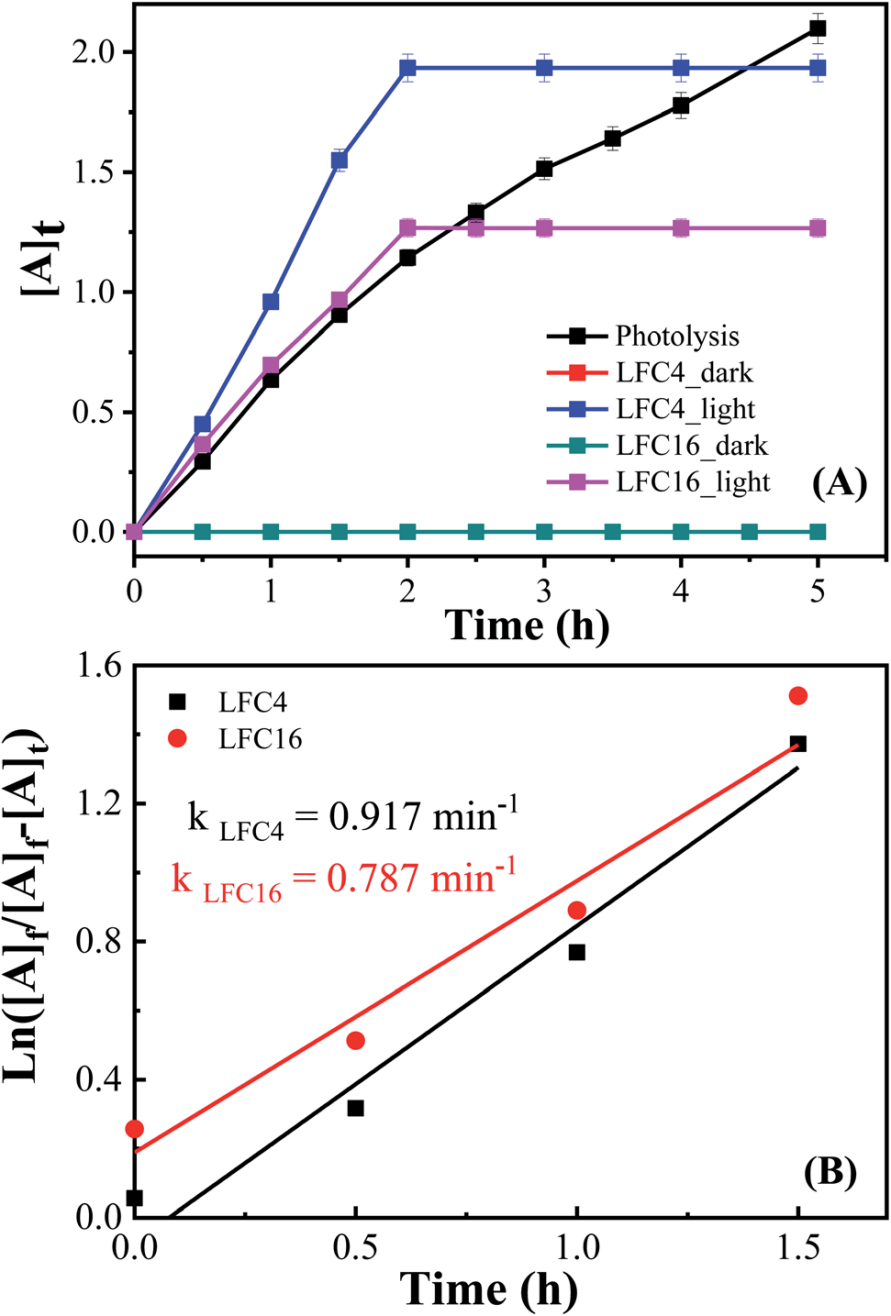
(A) Analysis of absorbance-time dependence in acetonitrile/water mixture in the presence of O_2_ atmosphere under dark and light conditions, and (B) integrated first-order kinetics for the photocatalytic conversion of OAP in an oxygen atmosphere.

Fig. 7 shows the analysis of absorbance-time dependence in acetonitrile/water mixture in the presence of oxygen air under dark and light conditions. No catalytic conversion was observed in dark conditions for both the LFC4 and LFC16 samples. In the absence of LaFeO_3_, the photolysis of OAP is enhanced in the presence of molecular oxygen within five hours. The LaFeO_3_ samples significantly enhance the chemical transformation of OAP to the hand-safe compound APX within two hours. The absorbance intensity of APX using LFC16 is lower than that of LFC4 due to the adsorption of the conversion product at the carbon residue-covered LaFeO_3_ NPs in the presence of molecular oxygen because the adsorption of the oxidative products is irreversible in the presence of molecular oxygen.^47^Fig. 7B represents the integrated pseudo-first-order kinetic for the photocatalytic conversion of OAP in an oxygen atmosphere. The calculated rate constant was found to be 0.917 and 0.787 min^−1^ for LFC4 and LFC16, respectively. Molecular oxygen enhanced the photocatalytic conversion of toxic OAP to safe compounds APX two times better than that in an inert atmosphere. Because of the utilization of photo-generated electrons in the reduction of oxygen to hydrogen peroxide, OAP is oxidized (Scheme 1). The LaFeO_3_ samples not only offer high catalytic conversion but are also easy to separate without losing their activity, as shown in Fig. S7.† Thus, the physicochemical features of LaFeO_3_ could be controlled using the economical sol–gel method and can be utilized efficiently as phenoxazinone synthase-like compound compared to previous studies, as shown in Table 1.

**Table 1:** Photocatalytic activity of LaFeO_3_ NPs toward OAP conversion compared to previous studies

Catalyst	Reaction condition	Rate constant	Ref.
Organo-tin(iv)–copper(i) cyanide coordination polymers [Ph_3_SnCu(CN)_2_L], where L = *trans*-1,2-bis(4-pyridyl)ethene (tbpe)	[OAP] = 0.015 M, methanol (5%) as a solvent and [catalyst] = 15 × 10^−5^ M, at 38 °C, *P*(O_2_) = 730 mm_Hg_ and pH = 9.0	1.2286 × 10^−5^ mol L^−1^ min^−1^	48
K_3_[Mn(C_2_O_4_)_3_]	[OAP] = 7.35 × 10^−4^ M in ethanol solution, at 25 °C	11.91 × 10^−4^ s^−1^	49
K_3_[Co(C_2_O_4_)_3_]		10.58 × 10^−4^ s^−1^	
K_2_[Cu(C_2_O_4_)_2_]		—	
2,2,6,6-Tetramethyl-1-piperidinyloxyl (TEMPO)	[OAP] = 0.11 M, [TEMPO] = 0.03 M, in methanol, under dioxygen at 50 °C	2.47 × 10^−4^ mol^−1^ dm^3^ s^−1^	50
Cobaloxime(ii) [Co(Hdmg)_2_L_2_], Hdmg^−1^ = dimethylglyoximato (1−), and L = PPh3, AsPh3, SbPh3	*P*(O_2_) = 1 atm, methanol as solvent at 25 °C	1.56 × 10^−2^ s^−1^	51
LaFeO_3_	3% acetonitrile as a solvent, [LaFeO_3_] = 0.0015 M, [OAP] = 0.5 mM, pH 7, at 25 °C under N_2_, and light intensity = 100 mW cm^−2^	7.2 × 10^−3^ s^−1^	This work
	3% acetonitrile as a solvent, [LaFeO_3_] = 0.0015 M, [OAP] = 0.5 mM, pH 7, at 25 °C under O_2_, and light intensity = 100 mW cm^2^	15.2 × 10^−3^ s^−1^	

## 4. Conclusions

Efficient photo nanozymes based on LaFeO_3_ NPs were developed for the conversion of highly toxic OAP to therapeutic reagent APX *via* phenoxazinone synthase mimicking activity. LaFeO_3_ NPs were synthesized by a facile sol–gel autocombustion method in the presence of different citric acid ratios. The photocatalytic conversion experiments of OAP were carried out in acetonitrile/water and ethanol/water mixtures in the absence and presence of molecular oxygen. Interestingly, the citric acid ratio controls the physicochemical characteristics of LaFeO_3_ nanoparticles in terms of the particle size, crystallinity, band gap, and functionality. With increasing citric acid ratio, a functional residual carbon enhanced the photocatalytic conversion of OAP. The presence of molecular oxygen also enhanced the photocatalytic rate by two times compared to that in the inert atmosphere. Based on our results, the holes and hydroxyl radicals are the active species for the photocatalytic oxidation of OAP over LaFeO_3_ NPs. The LaFeO_3_ samples showed low cost, excellent stability, and high activity for the photocatalytic transformation of OAP to APX and to clean-up the environment.

## Conflicts of interest

There are no conflicts to declare.

## Supplementary Material

RA-011-D1RA02295D-s001
