# Supplementary material for: Synthesis of highly crystalline LaFeO3 nanospheres for phenoxazinone synthase mimicking activity
Source: RSC Adv. 2021 May 17;11(29):17746–54. doi: 10.1039/d1ra02295d (PMC9033189; doi:10.1039/d1ra02295d)
Supplement: RA-011-D1RA02295D-s001 [file RA-011-D1RA02295D-s001.pdf]

Electronic Supplementary Material (ESI) for RSC Advances.
This journal is © The Royal Society of Chemistry 2021

Electronic Supporting information

Synthesis of highly crystalline $LaFeO_3$ nanospheres for phenoxazinone synthase mimicking activity

Mohamed Khairy,* Abdelrahman H. Mahmoud, and Kamal M. S. Khalil*

Chemistry Department, Faculty of Science, Sohag University, 82524.

S1. Calculation of CB and VB potentials for $LaFeO_3$.

La E_{IE} = 538.1 kj/mol ÷ 96.48 = 5.577 eV

E_{EA} = 48 kj/mol ÷ 96.48 = 0.498 eV

χ_{La} = 1/2(5.577+0.498) = 3.0375 eV

Fe E_{IE} = 762.5 kj/mol ÷ 96.48 = 7.9 eV

E_{EA} = 14.78 kj/mol ÷ 96.48 = 0.153 eV

χ_{Fe} = 1/2(7.9 eV +0.153) = 4.02 eV

O E_{IE} = 1313.9 kj/mol ÷ 96.48 = 13.618 eV

E_{EA} = 140.97 kj/mol ÷ 96.48 = 1.461 eV

χ_{O} = 1/2(13.618+1.461) = 7.54 eV

$\chi_{LaFeO3} = (3.0375\times 4.02\times (7.54)^3)^{1/5} = 5.54$ eV

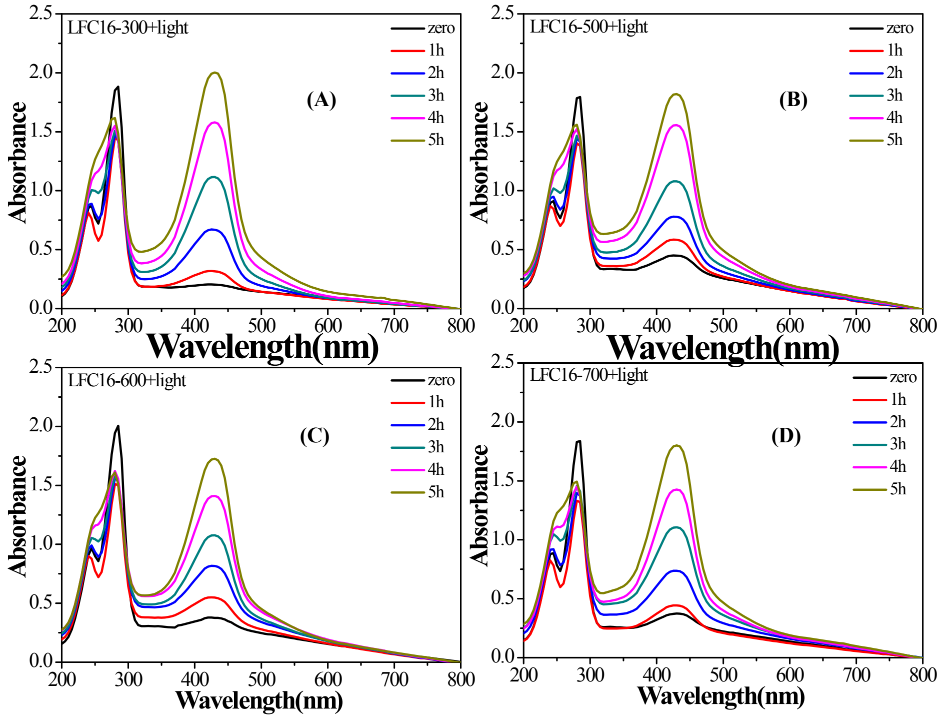

Figure S2. UV/Vis spectra of photocatalytic conversion of OAP over LFC16 at different calcination temperatures (A) 300 ^{o}C, (B) 500 ^{o}C, (C) 600 ^{o}C, and (D) 700 ^{o}C in ethanoic solution under nitrogen atmosphere.

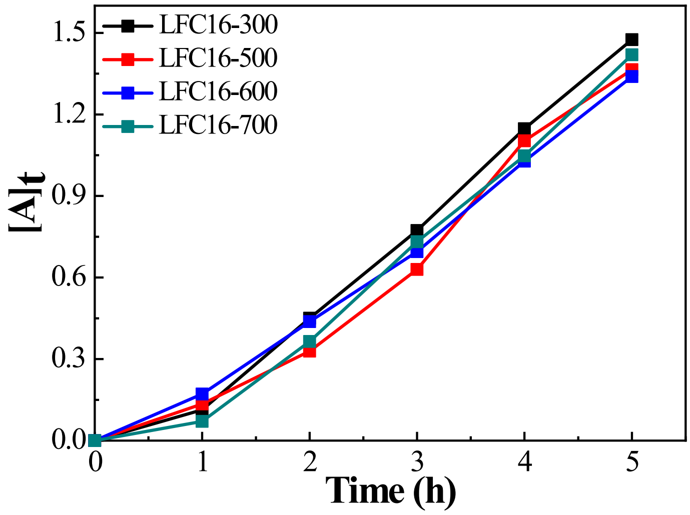

Figure S3. Analysis of absorbance time dependence in ethanol/water under nitrogen atmosphere using $LaFeO_3$ synthesized at different annealing temperatures under solar irradiation.

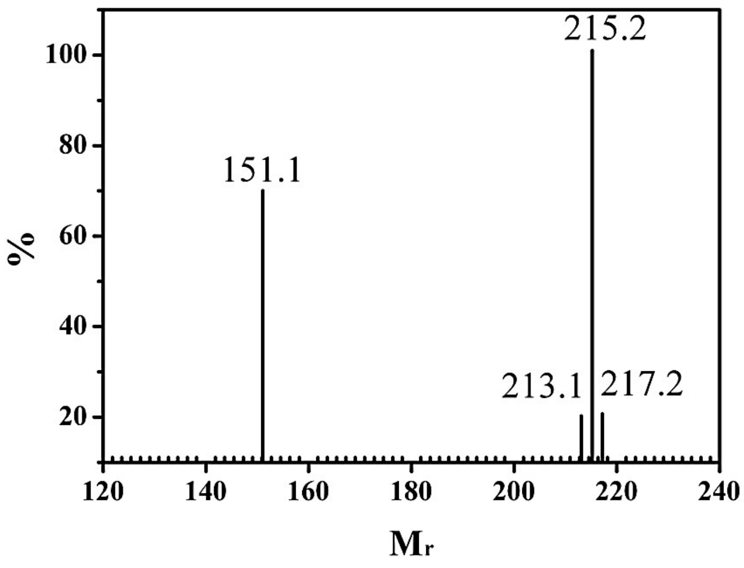

Figure S4. Mass spectra of isolated oxidation reaction products of *o*-aminophenol.

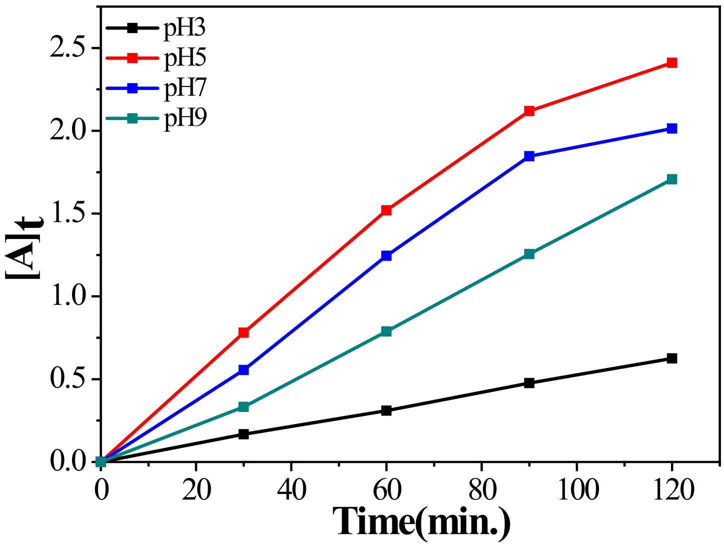

Figure S5. Effect of solution pH on the photocatalytic conversion of OAP to APX over LFC4 in acetonitrile/water solvent under nitrogen atmosphere under solar irradiation.

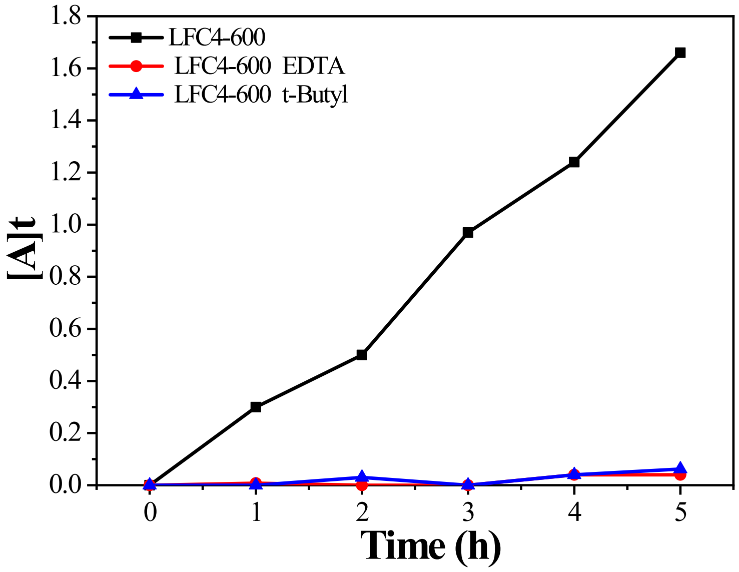

Figure S6. Effect of scavenger on the photocatalytic conversion of OAP to APX over LFC4-600 in acetonitrile/water solvent under nitrogen atmosphere under solar irradiation.

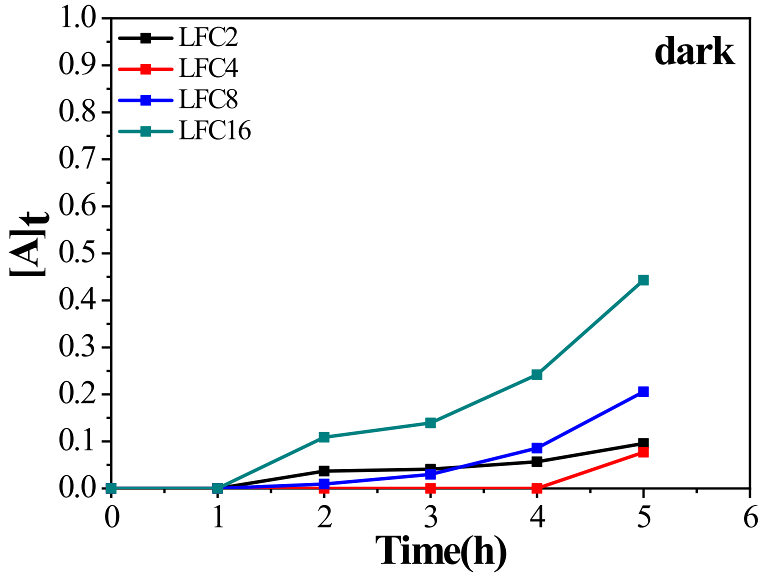

Figure S7. Analysis of absorbance time dependence in acetonitrile/water under nitrogen atmosphere using $LaFeO_3$ synthesized in dark.

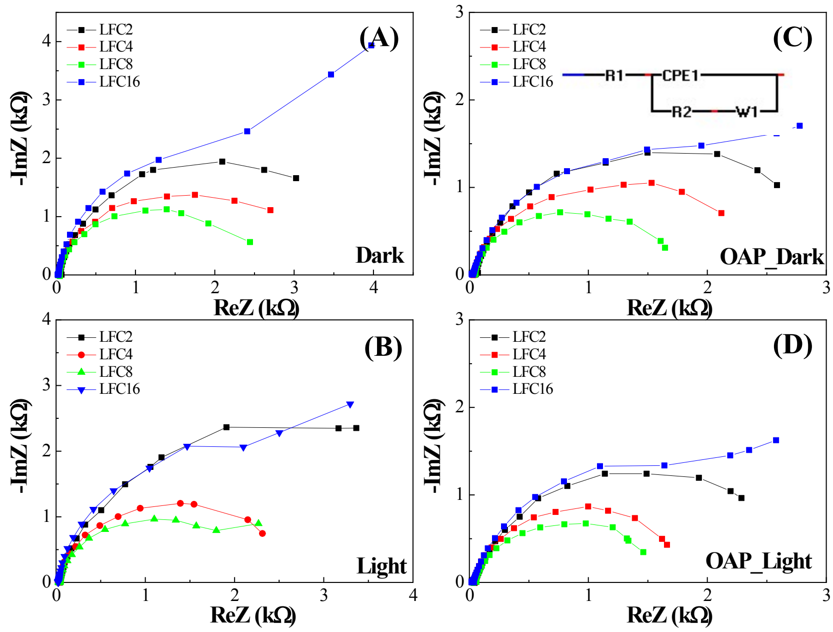

Figure S8. Nyquist plot of $LaFeO_3$ in (A, and B) absence and (C, and D) presence of 0.5 mM OAP in 0.1 M Na_2SO_4 at E_{DC} = 0.5 V vs. Ag/AgCl, E_{AC} = 10 mV (peak to peak) and frequency range of 100 kHz to 3 Hz.

Table S8. Electrochemical impedance analysis of $LaFeO_3$ samples.

	LFC2				LFC4				LFC8				LFC16			
	**Dark**	**Light**	**OAP Dark**	**OAP Light**	**Dark**	**Light**	**OAP Dark**	**OAP Light**	**Dark**	**Light**	**OAP Dark**	**OAP Light**	**Dark**	**Light**	**OAP Dark**	OAP Light
**R1 (Ω)**	28	30	40	35	24	23	23	21	26	28	33	40	20	24	23	20
**R2 (kΩ)**	4	4	3.1	2.75	3.1	2.6	2.1	1.85	2.45	2.1	1.6	1.5	3.2	4.4	2.7	2.6
**W ($Ωs^{-1}$)**	17	80	3	22	17	9	25	20	13	9	13	8	185	60	70	60
**P1 (µF)**	0.02	0.01	0.016	07, .013	0.02	0.018	0.02	0.02	0.02	0.018	0.014	0.018	0.013	0.02	0.012	0.012
**n1**	0.93	0.9	0.93	0.88	0.93	0.93	0.91	0.89	0.93	0.93	0.9	0.89	0.97	0.93	0.91	0.9

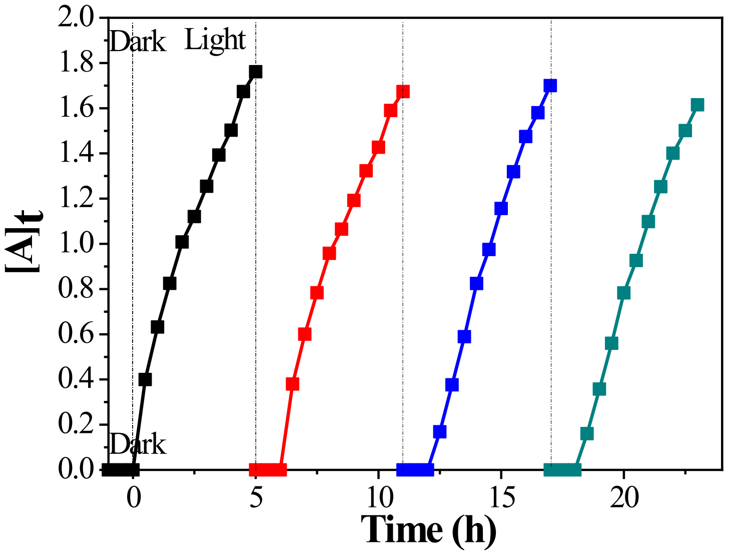

Figure S9. Reusability of LFC4 for photocatalytic conversion of OAP in acetonitrile/water under nitrogen atmosphere.
